# Plasma metabolomic profiling of dairy cows affected with ketosis using gas chromatography/mass spectrometry

**DOI:** 10.1186/1746-6148-9-186

**Published:** 2013-09-26

**Authors:** Hongyou Zhang, Ling Wu, Chuang Xu, Cheng Xia, Lingwei Sun, Shi Shu

**Affiliations:** 1 College of Animal Science and Veterinary Medicine, Heilongjiang Bayi Agricultural University, Daqing 163319, PR China

**Keywords:** Multivariable analysis, Clinical and subclinical ketosis, Gas chromatography/mass spectrometry, Plasma metabolome

## Abstract

**Background:**

Ketosis is an important problem for dairy cows` production performance. However, it is still little known about plasma metabolomics details of dairy ketosis.

**Results:**

A gas chromatography/mass spectrometry (GC/MS) technique was used to investigate plasma metabolic differences in cows that had clinical ketosis (CK, *n*=22), subclinical ketosis (SK, *n*=32), or were clinically normal controls (NC, *n*=22). The endogenous plasma metabolome was measured by chemical derivatization followed by GC/MS, which led to the detection of 267 variables. A two-sample *t*-test of 30, 32, and 13 metabolites showed statistically significant differences between SK and NC, CK and NC, and CK and SK, respectively. Orthogonal signal correction-partial least-square discriminant analysis (OPLS-DA) revealed that the metabolic patterns of both CK and SK were mostly similar, with the exception of a few differences. The development of CK and SK involved disturbances in many metabolic pathways, mainly including fatty acid metabolism, amino acid metabolism, glycolysis, gluconeogenesis, and the pentose phosphate pathway. A diagnostic model arbitrary two groups was constructed using OPLS-DA and receiver–operator characteristic curves (ROC). Multivariate statistical diagnostics yielded the 19 potential biomarkers for SK and NC, 31 for CK and NC, and 8 for CK and SK with area under the curve (AUC) values. Our results showed the potential biomarkers from CK, SK, and NC, including carbohydrates, fatty acids, amino acids, even sitosterol and vitamin E isomers, etc. 2-piperidinecarboxylic acid and cis-9-hexadecenoic acid were closely associated with metabolic perturbations in ketosis as Glc, BHBA and NEFA for dealing with metabolic disturbances of ketosis in clinical practice. However, further research is needed to explain changes of 2,3,4-trihydroxybutyric acid, 3,4-dihydroxybutyric acid, α-aminobutyric acid, methylmalonic acid, sitosterol and α-tocopherol in CK and SK, and to reveal differences between CK and SK.

**Conclusion:**

Our study shows that some new biomarkers of ketosis from plasma may find new metabolic changes to have clinically new utility and significance in diagnosis, prognosis, and prevention of ketosis in the future.

## Background

Ketosis is one of the most prevalent metabolic diseases of dairy cows during transition period [1]. Considering the serious consequences of ketosis such as fatty liver, abomasum displacement, infectious diseases, and reproductive diseases, this disease has become a health concern during the past decades [2,3]. Therefore, elucidating the pathogenesis of ketosis is of great importance to the monitoring, prevention, and early treatment of ketosis. To this end, there have been many reports about clinical pathological changes in dairy ketosis. β-hydroxybutyrate (BHBA) is widely considered the golden standard for diagnosing ketosis in dairy cows. In addition, blood glucose levels (Glc), total triglycerides (TG), nonesterified fatty acids (NEFA), and aspartate aminotransferase (AST) can also be analyzed to monitor ketosis-related complications [3-5]. However, to the best of our knowledge, the plasma metabolomic profiling of ketosis has not yet been clarified.

Metabolomics technology has proven to be a powerful tool for biomarker screening, disease diagnosis, and characterization of biological pathways in humans, rats, and cattle [6-10]. In view of the high efficiency of chromatographic separation and the sensitive detection of separated components, gas chromatography/mass spectrometry (GC/MS) is widely employed for the plasma metabolomic profiling of diseases, in combination with multivariate statistical analysis including principal component analysis (PCA), partial least square-discriminant analysis (PLS-DA), and orthogonal partial least square-discriminant analysis (OPLS-DA), etc. [11,12].

Although milk and plasma metabolome of ketosis has been reported using NMR technique [6,7], it is little known about metabolome profiles of clinical and subclinical ketosis. In this study, a metabolomics approach by GC/MS in conjunction with multivariate statistical analysis was developed to show alterations in plasma metabolite profiles of cows associated with normal or enhanced ketone body formation. Plasma samples from clinically normal cows or those with subclinical or clinical ketosis were profiled by GC/MS. Receiver operator characteristic curves (ROC) and OPLS-DA, which finds new variables with optimal discriminatory ability and small redundancy, were used to explore perturbations in metabolic patterns and potential biomarkers for clinical and subclinical ketosis.

## Results and discussions

### Comparison of demographics, clinical information and blood chemical parameters

Clinical data from normal cows (NC) or those with subclinical (SK) or clinical ketosis (CK) were collected to uncover any biochemical differences among the three groups shown in Table 1. Postpartum days, ages, parity, daily milk yield (MY), and body condition score (BCS) were comparable among the three groups of cows. There were significant differences in MY and BCS plasma Glc and BHBA concentrations among the three groups of cows, but not in plasma AST, CHO, and TG. Our results indicated that MY and BCS decreased significantly in dairy cows with ketosis, which is consistent with some reports that the affected cows often loss more milk yield and body weight [13,14].

**Table 1:** Main characteristics and blood biochemical parameters in normal control cows (NC), cows with subclinical ketosis (SK), and cows with clinical ketosis (CK)

**Parameters**	**CK**	**SK**	**NC**
Number	24	33	24
Days in milk	12±5	14±6	16±6
Age	3±1	3±1	4±2
Parity	2±1	2±1	2±1
MY (kg/d)	32.1±7.8^Aa^	35.2±7.2^b^	37.0±6.2^Cc^
BCS	3.05±0.31^a^	3.11±0.35 ^ab^	3.16±0.37^b^
Glc (mmol/L)	1.90 ± 0.70^A^	2.70±0.63^B^	3.37±0.58^C^
BHBA (mmol/L)	2.49±0.60^A^	1.22±0.17^B^	0.82±0.12^C^
CHO (mmol/L)	2.36±0.70	2.45±0.68	1.98±0.80
AST (U/L)	113.13±23.74	113.29±27.46	102.58±28.58
TG (mmol/L)	0.13±0.40	0.16±0.50	0.13±0.57

Data are shown as mean ± SD, p < 0.05 or 0.01 among CK, SK, and NC groups with different lowcase (a, b, c) or capital letters (A, B, C).

*MY* milk yield, *BCS* body condition score, *DMI* dry matter intake, *Glc* glucose, *BHBA* β-hydroxyl acid, *CHO* cholerol, *AST* aspartate aminotransferase, *TG* triglyceride.

All affected cows mainly presented low MY, loss of BCS, low blood Glc levels and high blood BHBA concentrations, which implies poor glucose regulation and lipid profile disorders. Although the above mentioned clinical biochemistry, such as Glc, BHBA, and AST levels, may to some extent reflect the metabolic state of ketosis, they only give limited information, as there are various metabolic abnormalities that can exist during ketosis progression, as well as other underlying metabolic changes associated with ketosis that can result in serious metabolic outcomes such as fatty liver and infectious diseases [1-3]. Thus, metabolomics may provide a more global and powerful approach for understanding the metabolic patterns and potential biomarkers of ketosis, and additionally, may shed light on the pathogenesis of ketosis in dairy cows.

### GC/MS metabolomic profiling

**Figure 1:**
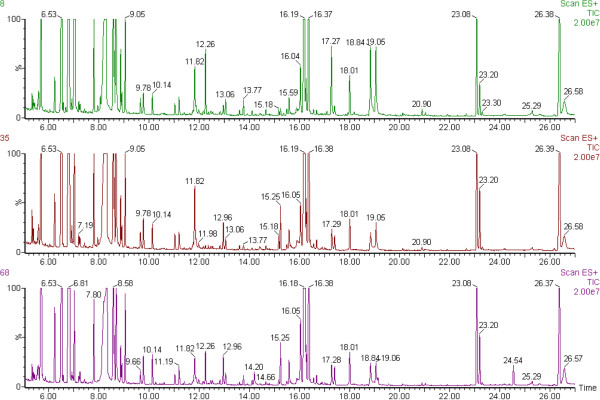
The representative GC/MS chromatograms of plasma samples from No. 8 of normal control (NC, purple peaks) cows, No. 35 of cows with subclinical ketosis (SK, brown peaks) and No. 68 of cows with clinical ketosis (CK, green peaks). X-axis is retention time (min); Y-axis is intensity of MS (%).

Figure 1 shows the three representative chromatograms of derived plasma samples from the three groups of cows, which had almost all same peaks among the three chromatograms, but there was also difference in peaks’ area among them. After mean-centering, UV-scaling, and deconvolution with AMDIS software, 40 of 267 variables were only significantly different between groups by multivariate statistical analysis, and then were identified as endogenous metabolites by NIST mass spectra library, and were categorized as carbohydrates, amino acids, fatty acids, sitosterol and vitamin E isomers, etc. (Table 2). Each of these is involved in multiple biochemical processes, especially those processes related to energy balance and lipid metabolism.

**Table 2:** Marker metabolites found in the GC/MS chromatograms of any two groups of CK, SK, and NC

**No**	**Compounds**^**a**^	**RT**^**b **^**(min)**	**Frags**^**c **^**(m/z)**	**CK *vs. *NC**			**SK *vs. *NC**			**CK *vs. *SK**			**Biochemical pathway**^**g**^
				**VIP**^**d**^	***P***^***e***^	**FC**^**f**^	**VIP**^**d**^	***P***^***e***^	**FC**^**f**^	**VIP**^**d**^	***P***^***e***^	**FC**^**f**^	
1	LA	5.68	147	1.88	2.62E-04	−0.79	2.13	4.90E-04	−0.73				Glycolysis;gluconeogenesis
2	GLCA	16.56	319	1.76	6.84E-04	−0.79	2.02	1.58E-03	−0.66				Pentose and glucuronate interconversions
3	L-ala	6.23	116	2.03	8.88E-05	−0.75	1.18	4.86E-02	−0.28	2.22	1.22E-03	−0.47	Alanine and aspartate metabolism
4	GA	5.87	147	1.89	1.53E-04	−0.56	2.12	5.94E-04	−0.48				Fatty acid metabolism
5	Ribitol	14.3	217	1.07	3.54E-02	−0.48	1.19	4.77E-02	−0.27				Glycolysis; Pentose phosphate Pathway
6	pGlu	11.83	156	1.54	2.83E-03	−0.43	1.34	1.77E-02	−0.29				Glutathione metabolism
7	Gal	16.03	73	1.83	9.86E-04	−0.38	1.85	3.02E-03	−0.61				Galactose metabolism
8	THBA	12.17	292	1.46	7.32E-04	−0.38	1.43	1.77E-02	−0.24				Fatty acid metabolism; Butanoate metabolism
9	Glc	16.21	319	1.66	2.98E-04	−0.31	1.77	2.78E-03	−0.22	2.26	2.15E-02	−0.14	Glycolysis/gluconeogenesis
10	Gly	9.05	174	1.45	1.62E-03	0.37	2.16	4.21E-05	0.45				Glycine, Serine and Threonine Metabolism
11	L-ile	8.87	158	1.67	1.72E-04	0.41	2.2	1.97E-05	0.44				Valine, leucine and isoleucine degradation
12	AABA	7.19	130	1.29	8.28E-03	0.53	1.94	1.89E-04	0.7				Fatty Acid Metabolism; Glycolysis; Glutamate metabolism;
13	AMA	11.19	218	1.35	2.58E-03	0.56	2.12	2.83E-05	0.67				dicarboxylic acid; malonate; biosynthetic formation of glycine
14	αTP	26.16	502	1.79	6.08E-03	0.57	1.11	5.02E-02	0.41				Antioxidant
15	Sitosterol	28.57	357	2.25	7.58E-07	0.70	1.37	6.92E-03	0.41	1.69	1.71E-02	0.29	Steroid biosynthesis;immunity
16	HMA	13.68	129	1.79	2.42E-04	0.82	1.39	6.73E-03	0.47				cholesterol synthesis and fatty acid mobilization
17	3HV	8.05	147	1.86	9.89E-05	0.91	1.8	7.83E-03	0.59	2.59	1.57E-03	0.31	Valine, leucine and isoleucine biosynthesis
18	PA	17.26	117	2.04	4.68E-05	1.00	2.05	1.25E-04	0.77				Fatty acid metabolism
19	HA	18.17	327	2.08	1.18E-05	1.09	2.24	2.94E-05	0.75	2.08	3.68E-02	0.34	Fatty acid metabolism
20	SA	18.71	341	2.26	5.60E-07	1.14	2.22	4.30E-05	0.76	2.34	8.85E-03	0.37	Fatty acid metabolism
21	BHBA	7.02	147	2.58	2.83E-10	1.33	3.06	2.43E-10	0.81	3.32	4.49E-05	0.53	Synthesis and degradation of ketone bodies
22	T-9-OA	18.82	339	2.09	1.76E-06	1.39	2.45	3.75E-07	1.06				Fatty acid metabolism
23	MA	15.36	285	1.73	7.58E-05	1.58	2.22	4.87E-06	1.14				Fatty acid metabolism
24	C-9-HA	17.09	311	1.68	9.90E-05	1.66	2.15	6.81E-06	1.28				Fatty acid metabolism
25	2PC	10.23	156	2.19	4.12E-06	1.79	2.77	1.53E-09	1.78				**Lysine metabolism**
26	L-ser	9.77	204	1.34	1.43E-02	−0.49				2.37	3.36E-04	−0.43	Glycine, serine, and threonine metabolism
27	GABA	11.91	84	1.73	2.91E-04	−0.71				2.27	8.44E-04	−0.48	Fatty acid metabolism; Glycolysis; Glutamate metabolism; Pyruvate metabolism
28	Melibiose	24.37	204	1.05	2.19E-02	−0.66							Arginine and proline metabolism
29	Erythritol	11.72	147	1.1	1.75E-02	−0.43							Galactose metabolism
30	3HIV	7.69	75	1.05	1.24E-02	−0.26							Pentose phosphate pathway
31	2Me3HB	7.53	117	1.34	5.91E-03	0.36							Valine, leucine and isoleucine biosynthesis
32	Xylitol	14.11	307	1.2	5.67E-03	0.42							Fatty acid metabolism; ketogenesis
33	4HYP	11.88	230				1.61	5.97E-03	0.47	1.3	2.90E-02	−0.24	Glycolysis; Pentose phosphate Pathway
34	L-orn	15.17	142				1.16	6.00E-02	−0.68				Urea Cycle; D-arginine and D-ornithine metabolism
35	MMA	8.53	319				1.33	1.83E-02	−0.39				dicarboxylic acid;malonate
36	2-KG	12.47	73				1.17	4.48E-02	−0.26				TCA Cycle; Glycolysis
37	L-leu	8.57	158				1.28	1.84E-02	0.19				Valine, leucine, and isoleucine metabolism
38	Citrate	15.24	73							1.72	7.87E-03	−0.59	TCA Cycle
39	DHBA	10.7	73							2.28	1.75E-02	−0.29	Fatty acid metabolism; Butanoate metabolism
40	L-pro	8.91	142							1.53	1.43E-02	−0.22	Arginine and proline metabolism

^a^Significant changes in the levels of corresponding metabolites between arbitrary two groups by the ANOVA and t-test (*P* < 0.05).

*2PC* 2-piperidinecarboxylic acid, *C-9-HA* cis-9-hexadecenoic acid, *MA* myristic acid, *T-9-OA* Trans-9-octadecenoic acid, *BHBA* 3-hydroxybutyric acid, *SA* stearic acid, *HA* heptadecanoic acid, *PA* palmitic acid, *3HV* 3-hydroxyvaleric acid, *HMA* 3-hydroxy-3-methylglutaric acid, *αTP* α-tocopherol, *AMA* aminomalonic acid, *AABA* α-aminobutyric acid, *L-ile* l-isoleucine, *Gly* glycine, *Glc* Glucose, *THBA* 2,3,4-trihydroxybutyric acid, *Gal* galactose, *pGlu* pyroglutamic acid, *GA* glycolic acid, *L-ala* l-alanine, *GLCA* glucuronic acid, *LA* lactic acid, *GABA* 4-aminobutyric acid, *L-ser* l-serine, *4HYP* 4-hydroxyproline, *3-HIV* 3-hydroxyisovaleric acid, *2Me3HB* 2-methyl-3-hydroxybutyric acid, *L-orn* l-ornithine, *MMA* methylmalonic acid, *2-KG* 2-ketoglutaric acid, *L-leu* l-leucine, *Cit* citrate, *DHB* 3,4-dihydroxybutyric acid, L-pro, l-proline.

^b^Retention time.

^c^Peaks in total ion chromatograms (TICs) are numbered according to their retention time. The identification of a metabolite is based on National Institute of Standards and Technology (NIST08) mass spectra database according to the match of masses (*m/z*) between the interested peak’s fragmentation pattern and that from the standard database.

^d^Variable importance in the projection (VIP) was obtained from OPLS-DA model with value higher than 1.0.

^e^The *p*-value calculated from two-tailed student's *t* test.

^f^Fold change (FC) was calculated as binary logarithm of average mass response ratio between two groups, where the positive value means that the average mass response of the metabolite in the former is larger than that in the latter and vice versa.

^g^Information from KEGG database or NuGOwiki database.

**Figure 2:**
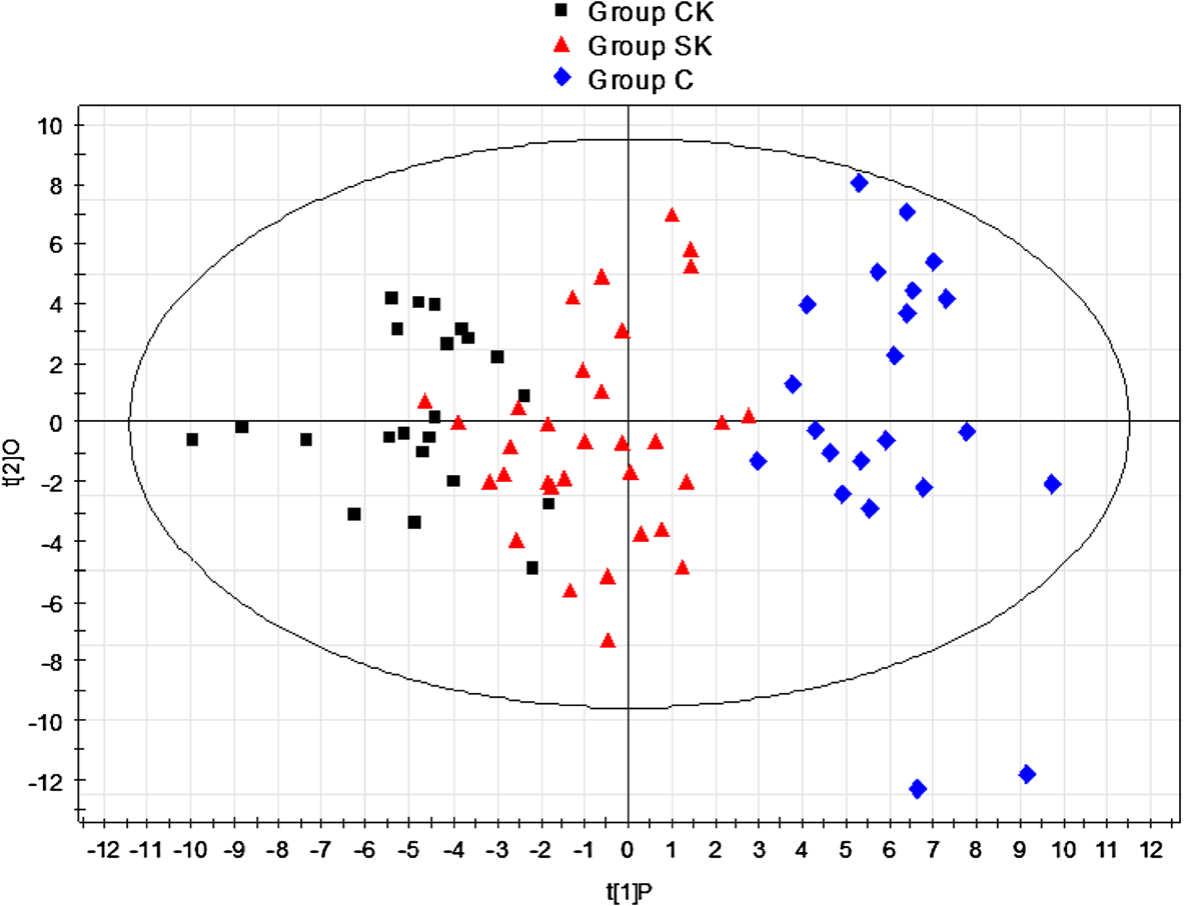
Score plot of OPLS-DA among three groups after excluding three outlier samples from Group CK, SK and NC respectively.

Since the metabolic profiles of CK and SK were similar, multivariate statistical analyses were used to explore biomarker candidates and disturbances in metabolic patterns of ketosis. PCA was used to examine the clustering of samples for differences in metabolic patterns among the three groups. The PCA score plot could not differentiate between CK, SK, and NC because the samples from the three groups scattered into each other; however, they could be successfully discriminated using the PLS-DA model (data not shown). Subsequently, OPLS-DA, which reduces dimensionality of the original data, was applied to explore metabolic disturbances in, NC, SK, and CK. The plot of PC1 (first principle component, t [1]P) versus O (orthogonal component, t [2]O) is shown in Figure 2. As illustrated in Figure 2, samples from the CK group lay on the left side of t [1]P, while samples from SK and NC lay in the middle and on the right side of t [1]P. In addition, the parameters of OPLS-DA, R^2^Y and Q^2^, were 0.828 and 0.693, respectively. R^2^Y showed the explanative ability of the model, and Q^2^ was the result of a seven-fold cross-validation, and showed the predictive ability of the model for metabolic profiling of the data [15,16]. These results indicate that the proposed metabolic process of ketosis was from NC to SK, and then to CK.

**Figure 3:**
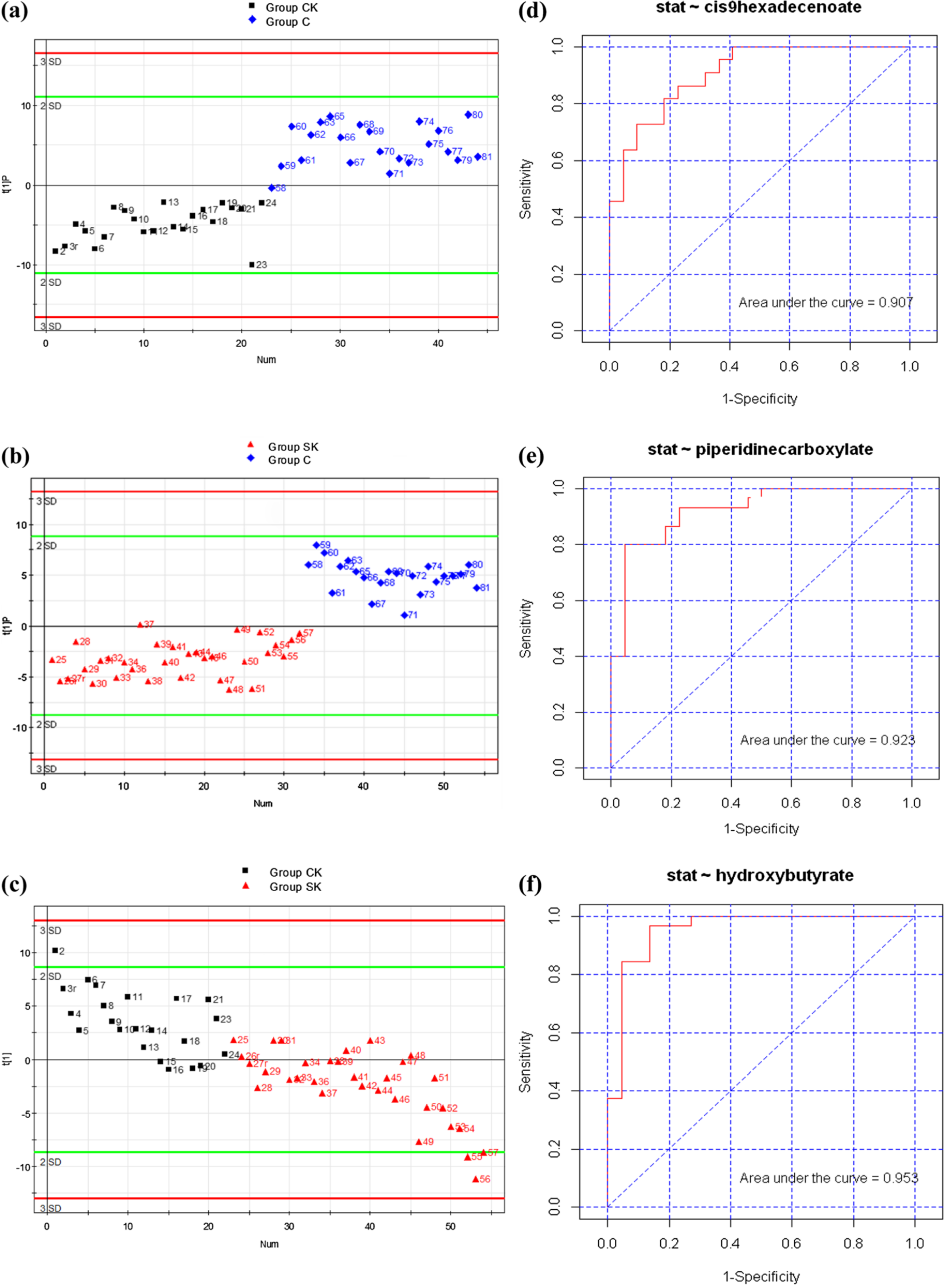
**The OPLS-DA scores plot and ROC plot of plasma obtained from arbitrary two groups. (a, b, c)** Score plot of OPLS-DA of using Principal Component 1 (PC1) in Group CK vs. NC, Group SK vs. NC, and Group CK vs. SK respectively; **(d, e, f)** ROC plot of cis-9-hexadecenoic acid, 2-piperidinecarboxylic acid, and 3-hydroxybutyric acid in the diagnosis in Group CK vs. NC, Group SK vs. NC, and Group CK vs. SK respectively.

To further investigate the metabolic status and potential biomarkers of two groups, samples from two groups were subjected to an OPLS-DA model. As can be seen in Figures 3 (a, b, c), samples from the corresponding two groups were distinctly separated on two sides of the PC1 baseline. Furthermore, the OPLS-DA parameters, R^2^Y and Q^2^, were 0.819 and 0.74 for CK vs. NC, 0.856 and 0.595 for SK vs. NC, and 0.931 and 0.301 for CK vs. SK, respectively. The results of the permutation test for the R^2^ and Q^2^ intercepts were 0.645 and −0.279 for CK vs. NC, 0.292 and −0.144 for SK vs. NC, and 0.323 and −0.105 for CK vs. SK, respectively. Thus, OPLS-DA was suitable for exploring differences between two groups in this study.

### Metabolic disturbances of ketosis

Table 2 lists 21 decreased metabolites from the three groups of cows, the majority of which were amino acids, carbohydrates, and their metabolites; the minority of which were carboxyl acids and hydroxyl acids from fatty acids metabolism and the tricarboxylic acid (TCA) cycle. Meanwhile it also shows the 19 increased metabolites, the majority of which were non-esterified fatty acids (NEFA), amino acids, and their metabolites; the minority of which were carbohydrates, and even sitosterol and vitamin E isomers, etc. These results suggest that metabolic disturbances of ketosis involve in multi-biochemical pathways such as glycolysis, gluconeogenesis, amino acids metabolism, fatty acids metabolism, pentose phosphate pathway.

In the current study, potential metabolites from arbitrary two groups were found to be significantly changed based on the largest VIP and a students t-test (*P* < 0.05). Table 2 summarizes 40 potential biomarkers that were differentially found in the CK, SK, and NC groups, which 25 metabolites were the same in both CK and SK, including 7 metabolites in CK vs. SK, indicating that CK and SK have rather similar pathogenesis.

**Figure 4:**
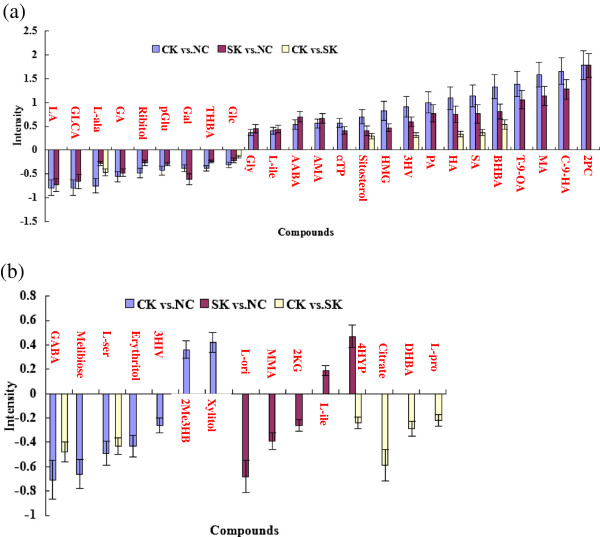
**Intensity of metabolites from CK compared to NC (blue column), SK compared to NC (red column), and CK compared to SK (yellow column). (a)** Expression intensity of 25 identical metabolites from CK vs. NC and SK vs. NC, including 7 metabolites from CK vs. SK. 2PC, 2-piperidinecarboxylic acid; C-9-HA, cis-9-hexadecenoic acid; MA, myristic acid; T-9-OA, Trans-9-octadecenoic acid; BHBA, 3-hydroxybutyric acid; SA, stearic acid; HA, heptadecanoic acid; PA, palmitic acid; 3HV, 3-hydroxyvaleric acid; HMA, 3-hydroxy-3-methylglutaric acid; VE, vitamin E (α-tocopherol); AMA, aminomalonic acid; AABA, α-aminobutyric acid; L-ile, l-isoleucine; Gly, glycine; Glc, Glucose; THBA, 2,3,4-trihydroxybutyric acid; Gal, galactose; pGlu, pyroglutamic acid; GA, glycolic acid; L-ala, l-alanine; GLCA, glucuronic acid; LA, lactic acid. **(b)** Expression intensity of 15 different metabolites from three groups. GABA, 4-aminobutyric acid; L-ser, l-serine; 4HYP, 4-hydroxyproline; 3-HIV, 3-hydroxyisovaleric acid; 2Me3HB, 2-methyl-3-hydroxybutyric acid; L-orn, l-ornithine; MMA, methylmalonic acid; 2-KG, 2-ketoglutaric acid; L-leu, l-leucine; Cit, citrate; DHB, 3,4-dihydroxybutyric acid; L-pro, l-proline.

Figure 4a shows that nine of the 25 metabolites; namely, lactic acid (LA), glucuronic acid (GLCA), l-alanine (L-ala), glycolic acid (GA), ribitol, pyroglutamic acid (pGlu), galactose (Gal), 2,3,4-trihydroxybutyric acid (THBA), and glucose (Glc), decreased from low to high in both CK and SK compared to NC. With the exception of L-ala in CK vs. SK and Gal in SK vs. NC, fold changes of other decreased metabolites are in turn low from CK vs. NC to SK vs. NC, and CK vs. SK. LA, L-ala, and pGlu from glucogenic amino acids and THBA from glucogenic and ketogenic threonine, which may be converted into glucose in liver by gluconeogenesis [17-20]. Low pGlu values may indicate glutathione deficiency due to oxidative stress [17]. Thus, gluconeogenesis decreased in both CK and SK because of a decrease in these glucogenic amino acids. In addition, GLCA, ribitol, Gal, and Glc can play a role in energy metabolism by entering glycolysis via the pentose phosphate pathway [21-24]. Decreased ribitol levels may be related to riboflavin deficiency [22]. Vitamin C may also be involved in energy metabolism due to GLCA, which is derived from vitamin C [21]. Therefore, a low level of plasma carbohydrates may play an important role in the development of ketosis, and low vitamin C and B2 levels, as well as oxidative stress, may also be contributing factors. It is also worth noting that a decrease in GA may reduce milk components in both CK and SK, since GA is used to synthesize milk fat or lactose [25]. GLCA and LA may play important roles in maintaining energy balance because they were maximally down-regulated in both CK and SK. In brief, our study suggests that ketosis is closely associated with disturbances in carbohydrate metabolism due to hypoglycemia and a lack of glucogenic precursors such as LA and L-ala. Vitamin C and B2 deficiencies, oxidative stress, and low GLCA and LA, may also contribute to the development of this disease.

Figure 4a also shows that 16 of 25 metabolites increased in both CK and SK compared to NC. With the exception of aminomalonic acid (AMA), α-aminobutyric acid (AABA), l-isoleucine (L-ile), and glycine (Gly), fold changes of other metabolites increased in turn from CK vs. NC to SK vs. NC, and CK vs. SK. These metabolites were mainly increased 3-hydroxybutyric acid (BHBA) and nonesterified fatty acids (NEFAs), including palmitic acid (PA), heptadecanoic acid (HA), stearic acid (SA), trans-9-octadecenoic acid (T-9-OA), myristic acid (MA), cis-9-hexadecenoic acid (C-9-HA), which belong to the families of ketone bodies, long chain unsaturated fatty acids, and saturated acids [1,26], confirming that a great amount of fat mobilization resulting from hypoglycemia may cause ketosis. In addition, some up-regulated amino acids and their catabolic products, such as L-ile, a glucogenic and ketogenic amino acid [25]; Gly, biosynthesized from serine [27]; AMA, a constituent of proteins before hydrolysis [28]; and 2-piperidinecarboxylic acid (2PC), a metabolite of the lysine metabolism [29]; suggest that proteolysis increases to meet body energy demand in both CK and SK. Since 2PC up-regulation was highest in both CK and SK, it may play an important role in ketone body synthesis as a catabolic product of ketogenic lysine [29]. In addition, the up-regulation of other metabolites, such as 3-hydroxyvaleric acid (3HV), 3-hydroxy-3-methylglutaric acid (HMG), and AABA, implicated abnormal metabolic changes or functional abnormalities in both CK and SK, since high 3HV concentrations can be found in methylmalonic acidemia [30], increased HMG values may be caused by decreased coenzyme Q10 synthesis [31], and elevated AABA levels can be indicative of liver disease [32]. However, it still remains unknown whether they play roles in the development of ketosis. Interestingly, we observed high sitosterol and vitamin E isomers in sick cows. In view of the anti-inflammatory property of sitosterol and the anti-oxidative role of α-tocopherol [33,34], those cows can not effectively utilize them, rendering them susceptible to infectious diseases and oxidative stress. However, it might be that the host responds to sickness by increasing those metabolites. It might be also both ways. Therefore, Need more search to see why they are high.

Although CK and SK had many of the same metabolites in both CK and SK, some different metabolites were also found between the two groups, as is shown in Figure 4b. Specifically, seven different metabolites increased in CK vs. NC, including 2-methyl-3-hydroxybutyric acid (2Me3HB) is a metabolite from isoleucine catabolism, β-oxidation of fatty acids and ketogenesis [25], and xylitol, is a precursor of xylulose 5-phosphate, and an intermediate of the pentose phosphate and glycolytic pathways [35]. The decreased metabolites included 3-hydroxyisovaleric acid (3HIV) from catabolism of the ketogenic amino acid leucine [36], 4-aminobutyric acid (GABA) from L-glutamic acid catabolism [37], melibiose from galactose metabolism [25], erythritol, a precursor of fructose 6-phosphate [38], and l-serine (L-ser), a glucogenic and ketogenic amino acid [39]. The results suggest that these carbohydrates and amino acids may be important factors in the development of CK. In addition, five different metabolites were up-regulated in SK compared to NC, and included 4-hydroxyproline (4-HYP) from proline hydroxylation [40], and l-leucine (L-leu), which is a ketogenic amino acid [19]. Down-regulated metabolites between the two groups included 2-ketoglutaric acid (2-KG), which is involved in the TCA cycle [41], l-ornithine (L-ori), which participates in the urea cycle [42], and methylmalonic acid (MMA), which is from a *C*-methylated derivative of malonate [43]. These results indicate that SK may disturb the TCA cycle, urea cycle, and odd-numbered fatty acid metabolism. Therefore, a few different metabolites had been found in CK or SK in this study, suggesting that they may play certain roles in identifying development process or types of ketosis.

Finally, Figure 4b shows the six metabolites that decreased in CK compared to SK; namely, GABA; L-ser; 4HYP; citrate; 3,4-dihydroxybutyric acid (DHB); l-proline (L-pro). Citrate is an intermediate in the TCA cycle [44]. DHB may be a product of amino acid catabolism [45]. L-pro is an important component of collagen and is derived from the amino acid l-glutamate [46]. Therefore, a decrease of the above mentioned metabolites, especially citrate, can further aggravate ketosis due to interruption of TCA cycle and lack of amino acids. Our research may shed light on the clinical potential of some potential metabolites in the development of ketosis.

In Table 2, long or medium-chain fatty acids and hydroxyl acids such as C-9-HA, MA, BHBA, and 2PC, etc. increased significantly in SK and CK compared with NC while metabolites such as 3-HV and 3-HIV changed differently in SK and CK group. The decreased metabolites were mainly some compounds such as LA, L-ala, GLCA, GA, Gal, Glc, etc., which are relative to glycolysis and TCA cycle. Thus, those metabolites may become potential biomarkers for diagnosing ketosis including SK and CK in dairy cows.

However, there were less differential metabolites in CK vs SK groups. Long or medium-chain fatty acids and hydroxyl acids such as HA, SA, BHBA, 3-HV ect. increased significantly in CK compared with SK, but amino acids like L-ala, L-ser, L-pro, 4-HYP, and GABA & citrate and DHBA decreased significantly. Therefore, those metabolites may be potential biomarkers for distinguishing the serious degree of ketosis in dairy cows.

In general, many metabolic pathways are disturbed in the development of ketosis. The supply of amino acids for gluconeogenesis decreases because there is a negative energy balance (NEB) in both CK and SK. In the meantime, the huge energy requirement for cow milk production after calving causes increased availability of fatty acids for oxidation, which displaces glucose as the oxidative fuel. In NEB situations, hypoglycemia activates fat mobilization, leading to NEFAs accumulation in liver, production of advanced glycolic end products, and increased oxidative stress, which result in many metabolic dysfunctions. The role of certain intermediates like THBA, DHBA, AABA, and MMA in the development of ketosis is worthy of further exploration in order to understand metabolic disturbances in ketosis.

### Diagnostic test and receiver–operator characteristic curves (ROC)

PCA, PLS-DA, and OPLS-DA, the most commonly used algorithms in metabolomics [33,46], were employed to process the GC/MS data. The OPLS-DA score plot showed that the two groups were scattered into two different regions using PC1 (Figures 3a, 3b, 3c). ROC analysis using the cross-validated predicted *Y* (predicted class) values was performed to validate the robustness of the OPLS-DA model. Sensitivity and specificity tradeoffs were summarized for each variable in Table 3, such as C-9-HA for CK vs. NC, 2PC for SK vs. NC, and BHBA for CK vs. SK, using the area under the curve (AUC), and were calculated using the trapezoidal rule (Figure 3d, AUC 0.907 in CK vs. NC; Figure 3e, AUC 0.923 in SK vs. NC; Figure 3f, AUC 0.953 in CK vs. SK). It has been reported that ROC analysis is able to determine easily ability for identifying disease at any cutoff. AUC value from ROC is usually between 1.0 and 0.5. AUC is more close to 1, the higher the accuracy test is, and the bigger the diagnostic value is , then the less false positive or negative ratio is [47,48]. In addition, the bigger positive likelihood ratio (+LR) is, the higher the true positive probability is [48,49]. According to AUC values and +LR of any two groups in Table 3, 31 metabolites for CK vs NC, 19 for SK vs NC, and 8 for CK vs SK may be potential biomarkers for identifying ketosis. Of course, those metabolites with high AUC values and high fold changes will be better potential diagnostic biomarkers for detection and classification of ketosis in the future.

**Table 3:** The potential diagnostic biomarkers of any two groups of CK, SK, and NC

**No**	**Compounds**^**a**^	**CK *vs *NC**		**SK *vs *NC**		**CK *vs *SK**	
		**LR+**^**b**^	**AUC**^**c**^	**LR+**^**b**^	**AUC**^**c**^	**LR+**^**b**^	**AUC**^**c**^
1	GA	0.25	0.122	0.38	0.176		
2	L-ala	0.06	0.149			0.30	0.216
3	L-ser					0.36	0.240
4	GLCA	0.19	0.153	0.05	0.19		
5	Erythritol	0.28	0.161				
6	Ribitol	0.26	0.171				
7	Glc	0.24	0.176	1.07	0.259		
8	LA	0.07	0.178	0.18	0.199		
9	Gal	0.34	0.184	0.42	0.22		
10	GABA	0.15	0.221			0.26	0.261
11	DHB					0.36	0.257
12	THB	0.38	0.231				
13	pGlu	0.47	0.269				
14	MMA			1.01	0.283		
15	Melibiose	1.05	0.277				
16	Citrate					0.42	0.281
17	3HV			2.32	0.79		
18	AABA			11.80	0.79		
19	L-leu	1.89	0.638				
20	Xylitol	3.16	0.752				
21	Gly	2.33	0.76	7.90	0.837		
22	2Me3HB	3.60	0.764				
23	AMA	2.57	0.773	3.09	0.822		
24	AABA	1.05	0.773				
25	αTP	2.37	0.783				
26	L-ile	3.16	0.814	3.32	0.827		
27	HMG	4.07	0.848				
28	Sitosterol	3.00	0.88			1.91	0.741
29	PA	6.35	0.897	5.29	0.838		
30	C-9-HA	3.81	0.909	2.75	0.842		
31	Hep	7.02	0.911	5.15	0.866		
32	MA	6.01	0.913	3.21	0.845		
33	SA	6.35	0.913	3.44	0.842		
34	2PC	4.97	0.918	7.78	0.923		
35	T-9-OA	4.21	0.924	3.58	0.868		
36	3HV	9.09	0.95			2.38	0.764
37	BHBA	10.22	0.977	8.13	0.965	5.11	0.953

^a^Abbreviations of compounds’ name are same as Table 2.

^b^+LR, positive likelihood ratio.

^c^AUC, area under the curve. If AUC value is more than 7.00 or less than 0.30, compound is considered to be a potential diagnostic biomarker of disease.

In this study, the detected metabolites that were differentially found between CK and SK were closely related to alterations in carbohydrates, fatty acids, amino acids, even sitosterol and vitamin E isomers, etc. Our study confirmed that BHBA is an excellent biomarker for ketosis including CK and SK, which has long been considered a gold indicator for diagnosing ketosis [2,3]. Hypoglycemia was the main biochemical characteristics in CK and SK (Table 1), which is in accordance with the metabolomics results in Table 2. Furthermore, C-9-HA, which belongs to the NEFA family, was significantly increased in CK compared to NC, an important indicator of the NEB and fat mobilization. However, Glc and the NEFA family could not become potential biomarkers for distingushing CK and SK. In addition, the increased 2PC in SK compared to NC, which has not been previously reported as a potential biomarker, suggested that some new biomarkers may play important roles in the pathogenesis and development of ketosis in dairy cows.

## Conclusion

The present study is an integrated analysis in dairy cattle ketosis based on plasma metabolomic profiling by GC/MS. We first discovered that 40 metabolites (i.e. fatty acids, amino acids, carbohydrates, and others) were differentially found among CK, SK and NC. This proved that metabolic patterns of ketosis could be reflected by metabolomics technology, which could, to some extent, reveal the development and progression of ketosis. To research ketosis, potential biomarkers of CK and SK could uncover the same or different modes of metabolites and metabolic pathways in the development and progression of ketosis. Furthermore, new potential metabolites could shed light on new strategies for the diagnosis, prognosis, and prevention of ketosis in the future.

## Methods

### Animals and sample collection

All experimental animals were treated according to the International Guiding Principles for Biomedical Research Involving Animals. Twenty-two cows with clinical ketosis (CK), 32 cows with subclinical ketosis (SK), and 22 normal control cows (NC) from an intensive 1000 dairy cattle farm in Mishan, Heilongjiang, China were used in this exploratory study. All cows were fed a total mixed ration (TMR) during the transition period. TMR consisted of 8.5 kg of concentrated feed, 18.5 kg of silage maize, 4 kg of alfalfa, and 0.35 kg of fat. TMR consisted of 55.60% dry matter (DM), 16% crude protein, 1.75 mcal/DM net energy for lactation (NEL), 5.60% fat, 39.10% neutral detergent fiber (NDF), 20.30% acid detergent fiber (ADF), 180 g Ca, and 116 g P. The cows were considered to have CK if they showed typical clinical symptoms including lack of appetite, apparent wasting, low milk yield, even nervous signs etc., and had high plasma BHBA concentrations ( > 1.60 mmol/L). If the cows had little clinical symptoms and high plasma BHBA concentrations (> 1.20 mmol/L), they were considered to have SK. If the cows had no clinical signs and normal plasma BHBA concentrations (< 1.00 mmol/L), they were considered normal controls (NC) [3,4]. Within 12 h of calving, whole first-morning blood samples were collected and immediately centrifuged at 1400 × g for 10 min at room temperature. The supernatants were aliquoted into Eppendorf tubes (1 mL plasma/tube) and stored at −80°C until analysis. Both clinical parameters and plasma metabolomics were measured for all experimental animals. Main characteristics and blood biochemical parameters, which included age, parity, milk yield (MY, kg/d), body condition score (BCS), blood glucose (Glc), β-hydroxaybutyric acid (BHBA), triglycerides (TG), aspartate aminotransferase (AST), and cholesterol (CHO), are shown in Table 1.

### Sample preparation

The sample preparation was modified according to literature [48,49] and described as follows. To an Eppendorf tube were added 50 μL of thawed plasma at room temperature, 10 μL ^13^C_6_-Leucine (0.5 mg/mL in water, as internal standard), and 180 μL cold methanol. The mixture was vigorously vortexed for 30 s and centrifuged at 16 000×g for 15 min at 4°C, and then 192 μL of the supernatant was transferred to a glass vial for drying under gentle nitrogen stream. A volume of 80 μL of methoxylamine hydrochloride in pyridine (20 mg/mL) was added to the residue, and the solution was strongly vortexed for 30 s and incubated at 37°C for 90 min. The sample was derivatized using 80 μL N,O-bis (trimethylsilyl)-trifluoroacetamide (BSTFA) with 1% trimethylsilyl chloride (TMCS) at 70°C for 60 min. To avoid decomposition, all derivatized samples were analyzed within 48 h by GC/MS (Agilent 7890A GC /5975C MS).

### Data preprocessing and multivariate statistical analysis

The extraction, alignment, deconvolution, and further processing of raw GC/MS data were referred to the protocols of literature [47-49], except the mass window was set to 70–600 m/z. The preprocessed data table, including observations (sample name), variables (rt_mz), and summarized peak area, was imported into Simca-P 11.0 software (Umetrics AB, Umea, Sweden) for multivariate statistical analysis, where mean-centering and UV-scaling were employed prior to PCA, PLS-DA, and OPLS-DA. Principal component analysis (PCA) was initially performed for visualizing the trends of samples in this study. To remove the noise, partial least square-discriminant analysis (PLS-DA) and orthogonal partial least square-discriminant analysis (OPLS-DA) were utilized to explore differences among groups. PLS-DA was used to model the metabolic profiling data among CK, SK, and NC. OPLS-DA filtered unrelated factors to CK, SK, and NC in the metabolic profiling. Model parameters, R^2^Y and Q^2^, were applied to evaluate model stability and the ability to explain and predict the raw data. In addition, the possibility of over-fitting was tested using the permutation test [11,12,15].

### Identification and qualification of differential metabolites

Differential metabolites (i.e., potential biomarkers) between arbitrary two groups were found by combining Variable Importance in Projection (VIP >1) values of the OPLS-DA model and students t-test (*P* <0.05). The structural qualification of potential biomarkers was performed according to the method of literature [5-7]. The automated mass spectral deconvolution and identification system (AMDIS, National Institute of Standards and Technology, Gaithersburg, MD) was first utilized to deconvolute mass peaks and search the self-constructed standard library by matching retention time and mass spectra pattern. The peaks which were not matched from standard library were introduced to the NIST MS 2.0 software for automatically searching from the NIST 08 library. The peaks with matching similarity larger than 80% were assigned as candidate compounds.

#### Statistical analysis

Variance was used to analyze the some information based on the diagnoses of CK, SK, and NC. All data processed by ANOVA (SPSS for Windows version 11.0) were presented as mean ± standard deviation (SD). *P* values of less than were considered to indicate a significant difference. In addition, the receiver operating characteristic (ROC) analysis was carried out using SPSS, and the AUC value, specificity, sensitivity were calculated to evaluate the diagnostic value of the potential biomarkers from the differential metabolites of the disease. *P* values of less than 0.05 were considered to indicate a significant difference [47-49].

## Abbreviations

AMA: Aminomalonic acid; AABA: α-aminobutyric acid; BHBA: 3-hydroxybutyric acid; C-9-HA: cis-9-hexadecenoic acid; DHBA: 3,4-dihydroxybutyric acid; GA: Glycolic acid; Gal: Galactose; Gly: Glycine; Glc: Glucose; GLCA: Glucuronic acid; GABA: 4-aminobutyric acid; HA: Heptadecanoic acid; HMA: 3-hydroxy-3-methylglutaric acid; LA: Lactic acid; L-ala: l-alanine; L-ile: l-isoleucine; L-leu: l-leucine; L-orn: l-ornithine; L-pro: l-proline; L-ser: l-serine; MA: Myristic acid; MMA: Methylmalonic acid; PA: Palmitic acid; pGlu: pyroglutamic acid; SA: Stearic acid; T-9-OA,THBA: 2,3,4-trihydroxybutyric acid; Trans-9-octadecenoic acid; 3HV: 3-hydroxyvaleric acid; αTP: α-tocopherol; 2-KG: 2-ketoglutaric acid; 2PC: 2-piperidinecarboxylic acid; 4HYP: 4-hydroxyproline; 3-HIV: 3-hydroxyisovaleric acid; 2Me3HB: 2-methyl-3-hydroxybutyric acid.

## Competing interests

There are no conflicts of interests.

## Authors’ contributions

HYZ performed the metabolomic samples preparation, data generation, analyzed and wrote the draft of the manuscript. CX participated in design of this study and helped to interpret the metabolomic data and analysis. CX and LW conceived of the study, participated in its design and coordination, helped to interpret the statistical analysis of the metabolomic data and draft the manuscript. LWS and SS did sample collection, sample preparation, facilitated sample collection, contributed to design of the study, provided knowledge in dairy metabolic biology and helped to draft the manuscript. All authors read and approved the final manuscript.
